# Umbilical cord-derived Wharton’s jelly for treatment of knee osteoarthritis: study protocol for a non-randomized, open-label, multi-center trial

**DOI:** 10.1186/s13018-021-02300-0

**Published:** 2021-02-18

**Authors:** Ashim Gupta, Nicola Maffulli, Hugo C. Rodriguez, Cassidy E. Lee, Howard J. Levy, Saadiq F. El-Amin

**Affiliations:** 1 BioIntegrate, Lawrenceville, GA USA; 2 Future Biologics, Lawrenceville, GA USA; 3 South Texas Orthopedic Research Institute (STORI Inc.), Laredo, TX USA; 4 Veterans in Pain (V.I.P.), Los Angeles, CA USA; 5 Department of Musculoskeletal Disorders, School of Medicine and Surgery, University of Salerno, Fisciano, Italy; 6 San Giovanni di Dio e Ruggi D’Aragona Hospital “Clinica Orthopedica” Department, Hospital of Salerno, Salerno, Italy; 7 Barts and the London School of Medicine and Dentistry, Centre for Sports and Exercise Medicine, Queen Mary University of London, London, UK; 8 School of Pharmacy and Bioengineering, Keele University School of Medicine, Stoke on Trent, UK; 9 School of Osteopathic Medicine, University of The Incarnate Word, San Antonio, TX USA; 10 Future Physicians of South Texas, San Antonio, TX USA; 11 El-Amin Orthopaedic and Sports Medicine Institute, 2505 Newpoint Pkwy, Suite 100B, Lawrenceville, GA 30043 USA; 12 Department of Orthopaedic Surgery, Lenox Hill Hospital, Northwell Health, New York, NY USA

**Keywords:** Umbilical cord, Wharton’s jelly, Knee osteoarthritis, Regenerative medicine, Biologics, Clinical trial, Extracellular vesicles, Exosomes, Growth factors, Hyaluronic acid

## Abstract

**Background:**

Osteoarthritis (OA) is the most common joint disorder in the USA, and knee OA has the highest prevalence. Inflammation and decrease in vascularization are key factors in the degeneration of articular cartilage and the associated pain and decrease in function. To combat this process, the use of biologics including umbilical cord-derived Wharton’s Jelly (UC-derived WJ) has grown. UC-derived WJ contains large quantities of regenerative factors, including growth factors (GFs), cytokines (CKs), hyaluronic acid (HA), and extracellular vesicles (EVs). The proposed study evaluates the safety and efficacy of intraarticular injection of UC-derived WJ for treatment of knee OA symptoms.

**Methods and analysis:**

This is a non-randomized, open-label, multi-center, prospective study in which the safety and efficacy of intraarticular UC-derived WJ in patients suffering from grade II/III OA will be assessed. Twelve patients with grade II/III OA who meet the inclusion and exclusion criteria will be recruited for this study which will be conducted at up to two sites within the USA. The participants will be followed for 1 s. Participants will be assessed using the Numeric Pain Rating Scale (NPRS), Knee Injury and Osteoarthritis Outcome Score (KOOS), 36-item short form survey (SF-36), Single Assessment Numeric Evaluation (SANE), physical exams, plain radiography, and Magnetic Resonance Observation of Cartilage Repair Tissue (MOCART) score for improvements in pain, satisfaction, function, and cartilage regeneration.

**Discussion:**

This prospective study will contribute to the limited amount of data on UC-derived WJ, particularly with regard to its safety and efficacy. The outcomes from this study will also lay the groundwork for a large placebo-controlled trial of intraarticular UC-derived WJ for symptomatic knee OA.

**Trial registration:**

ClinicalTrials.gov NCT04719793. Registered on 22 January 2021

## Background

Osteoarthritis (OA) affects approximately 30 million American adults aged 25–74 years, making it the most common joint disorder in the USA [1]. OA is characterized by degeneration of articular cartilage and secondary osteogenesis, with the earliest pathological changes seen in the articular cartilage [2]. Larger weight-bearing joints such as knees, hips, and the facet joints of the spine are OA most frequent targets [3, 4]. Of all the joints it affects, knee OA is the most prevalent with the number of adults suffering expected to reach 67 million by 2030 [5, 6].

While knee OA is a prominent cause of disability in adults, there is no clear etiology to explain its pathology. Knee OA has been suggested to be related to age, obesity, joint trauma, mechanical damage, gender, and other factors [7, 8]. The pathology of knee OA may be linked to degenerative lesions in cartilage secondary to inflammation associated with hyperplasia and chondrocyte apoptosis [9, 10]. Increasing age is linked to a reduction in subchondral blood vessels resulting in cartilage related physiological and biochemical anomalies [11]. Additionally, the inability of long-chain hyaluronic acid and polyglucose to generate chondrocytes results in local softening of articular cartilage, loss of elasticity, wear, and structural damage. This pathological process results in secondary joint fibrosis, stiffness, pain, and decreased function; leading to a poor quality of life [8, 11]. Knee OA treatment aims to decrease or eradicate pain, enhance or restore joint function, rectify any morphological or alignment defects, and improve quality of life.

Currently, there are various treatment options used in clinical practice to manage knee OA, including activity modification, physical therapy, pharmacological agents such as NSAIDs, corticosteroids, viscosupplementation, and narcotics. These treatment modalities have shown variable and limited clinical benefits and have potential side effects. When conservative measures fail, total knee replacement is usually recommended [12–20]. While total knee replacement (TKR) surgeries typically result in decreased pain, improved joint function, and reduced disability; complications, such as infection, persistent pain, and loss of motion may occur, and may require revision surgery. In addition, outcomes after TKR surgeries for patients with less severe knee OA (grades II and III) are worse compared to patients with grade IV OA (on Kellgren-Lawrence scale) [21–23]. An additional goal of non-operative therapy is to delay or even avoid surgical intervention. Decreasing the number of TKR surgeries will result in fewer revision surgeries, potentially saving patients from multiple costly surgeries and extensive rehabilitations, and decreasing the healthcare burden [21].

Over the last decade, the use of biologics for regenerative medicine applications has gained popularity [24–30]. Despite their increased use, there are inadequate studies evaluating the amount of growth factors (GFs), cytokines (CKs), hyaluronic acid (HA), and extracellular vesicles (EVs) including exosomes present in these products. Specifically, there is limited or no clinical literature assessing the safety and efficacy of UC-derived WJ products. We formulated an UC-derived WJ product and analyzed it for the presence of these factors. The vital elements of regenerative medicine, namely GFs, CKs, HA, and EVs, are all present in large quantities in the formulated WJ [31]. This study allowed us to characterize this novel WJ formulation prior to conducting clinical trials to determine the safety and efficacy—for regenerative medicine applications.

The goal of the proposed study is to evaluate the safety and efficacy of intraarticular injection of UC-derived WJ for treatment of knee OA symptoms. We hypothesize that the intraarticular injection of WJ is safe, and participants will show an improvement in their overall satisfaction, Numeric Pain Rating Scale (NPRS), Knee Injury and Osteoarthritis Outcome Score (KOOS), and cartilage formation over a period of 1 year compared to the baseline visit. Our null hypothesis is that there is no difference between baseline and after-treatment time-points over a period of 1 year.

## Methods and analysis

This study protocol is reported in accordance with the Standard Protocol Items- Recommendations for Intervention Trials (SPIRIT) criteria [31, 32]. The complete SPIRIT checklist can be found in Supplementary data.

### Study design

**Fig. 1:**
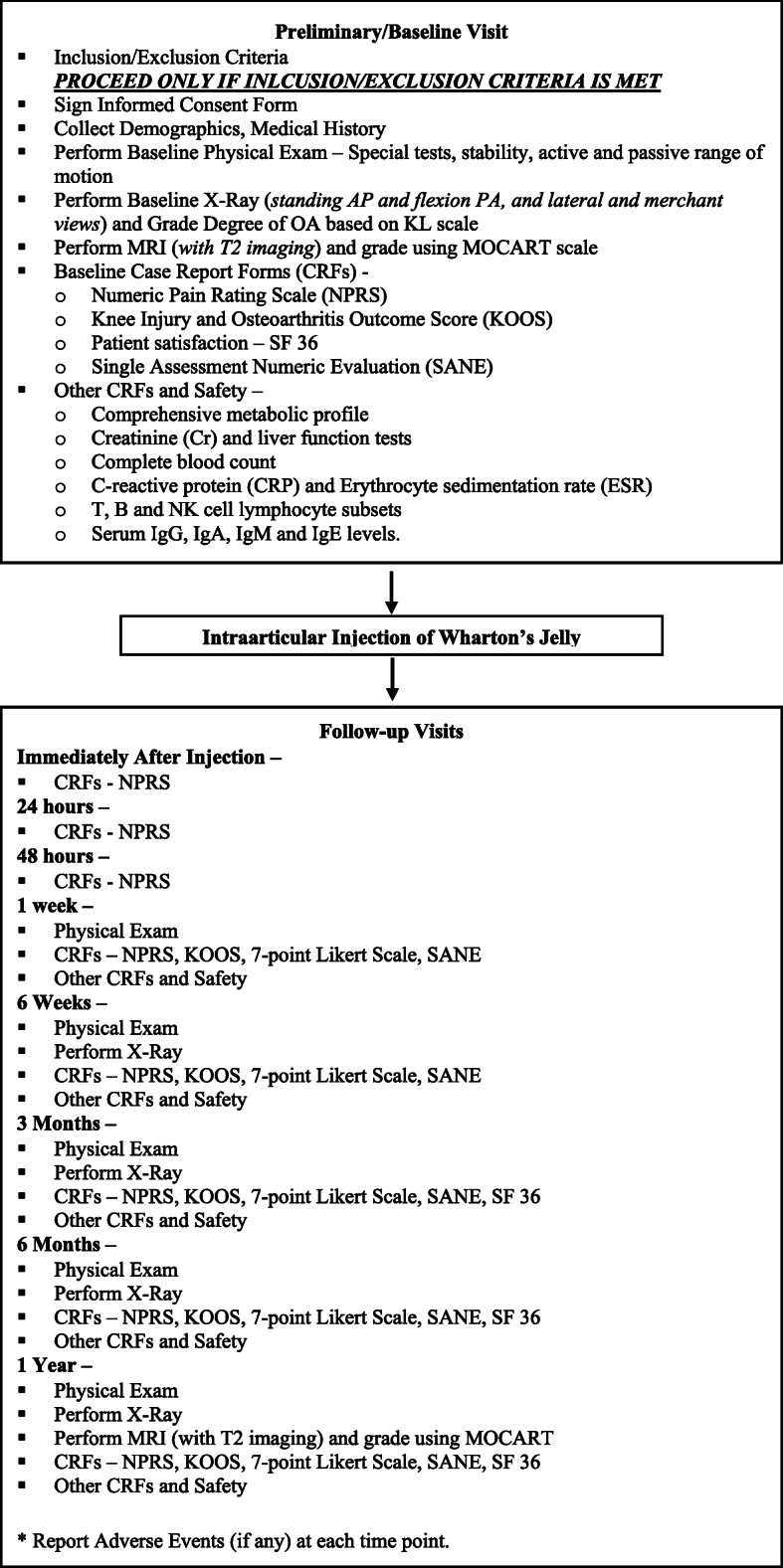
Summary of trial design

**Fig. 2:**
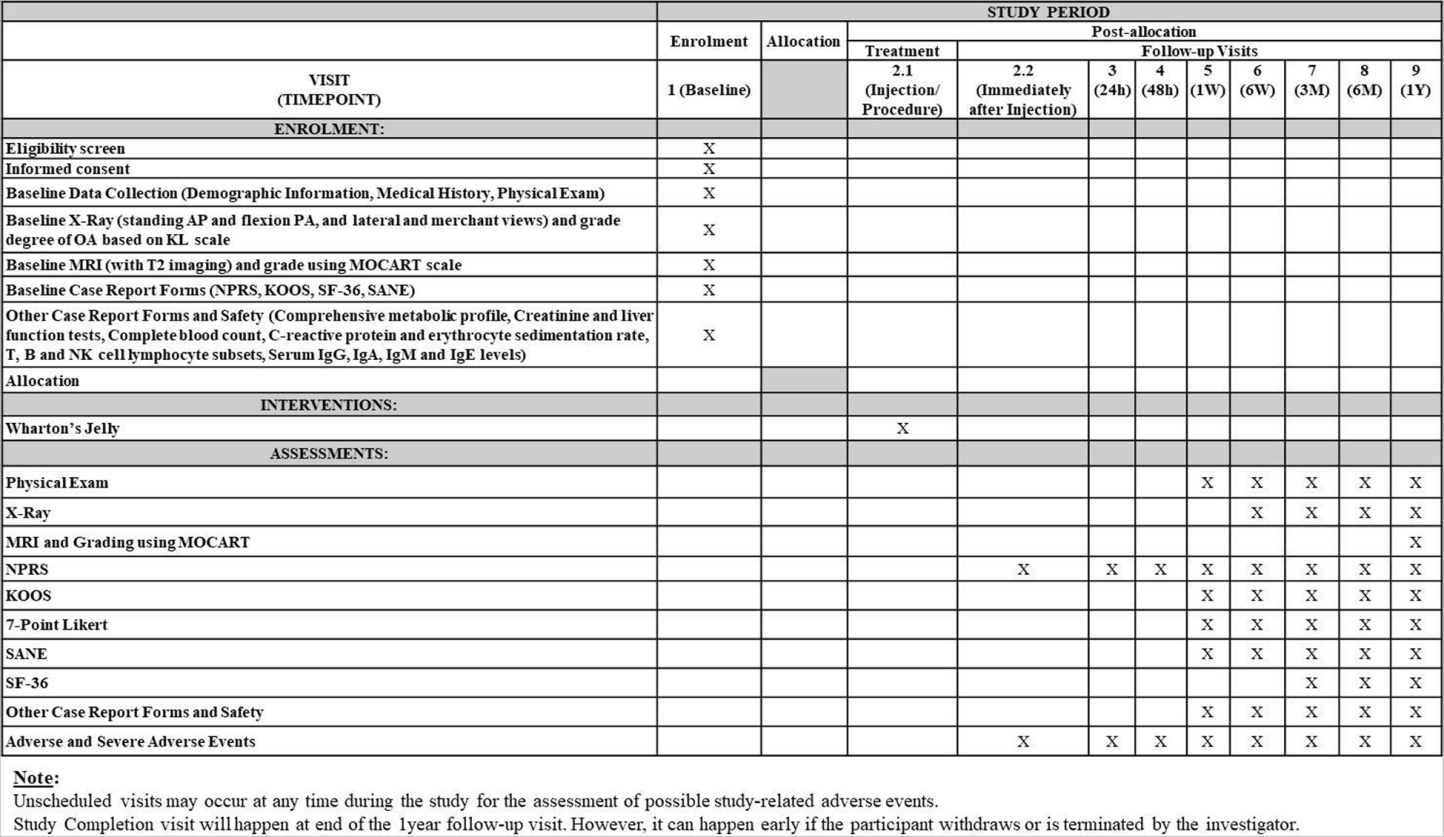
Standard protocol items: recommendations for interventional trials (SPIRIT) flowchart

Twelve patients with grade II/III OA who meet the inclusion and exclusion criteria will be recruited for this non-randomized, open label, multi-center, prospective study. The study will be conducted at two sites within the USA, and the patients will be followed for 1 year, with an expected duration of 15 months (Figs. 1 and 2). Figure 2 depicts the schedule for enrolment, intervention and assessment according to the SPIRIT guidelines.

### Inclusion criteria

Patients who are 18 years or older with a body mass index (BMI) of < 40 kg/m^2^ and a diagnosis of mild to moderate (grade II/III) OA in only one knee on the KL grading scale will be recruited. Patients must also meet the following criteria:

1. Pain score of 4 or more on the NPRS
2. Willing and capable of giving written informed consent to participate
3. Willing and capable of complying with study-related requirements, procedures, and visits
4. Female patients must be abstinent, surgically sterilized, or postmenopausal
5. Premenopausal females with negative pregnancy test, and who does not anticipate pregnancy and will actively practice an accepted contraceptive method for a duration of the study
6. Males with premenopausal female partners will take contraceptive measures for the duration of the study

### Exclusion criteria

Patients who have taken any pain medications including non-steroidal anti-inflammatory drugs (except acetaminophen) within 15 days of the study injection date or that regularly use anticoagulants, have a substance abuse history and/or fail to agree not to take any knee-symptom modifying drugs during the course of the study without proper reporting to the site PI and study team will not be eligible to participate. Patients must also not meet the following criteria:

1. Evidence of pathological knee laxity or instability on physical exam
2. History of intraarticular injection of any drug including corticosteroids or viscosupplementation in the index knee within the last 3 months
3. Knee surgery on the index knee within the last 6 months
4. Traumatic injury to the index knee within the last 3 months
5. Planned elective surgery during the course of the study
6. Organ or hematologic transplantation history, rheumatoid arthritis, or other autoimmune disorders
7. Immunosuppressive medication/treatment
8. Diagnosis of non-basal cell carcinoma within the last 5 years
9. A knee infection or use of antibiotics for a knee infection within the last 3 months
10. Participation in another clinical trial or treatment with any investigational product within the last 30 days prior to inclusion
11. Female patients who are breast feeding or are pregnant or desire to be pregnant during the course of the study
12. Contraindications to plain radiography or MRI imaging
13. Serious neurological, psychological or psychiatric disorders
14. Other medical conditions determined by the site principal investigator as interfering with the study
15. An injury or disability claim under current litigation or pending or approved workers’ compensation claim

Participants will have the opportunity to voluntarily withdraw from the study at any time without any sanction or affect to their access to other treatments. The participation of a patient in the study may be terminated if continued participation is not in the subject’s best interest based on standard medical practice by the PI. Any participant with any adverse events (AEs) regardless of whether it is related to the treatment can withdraw voluntarily from the study.

### Study intervention

After patients are determined to be eligible for the study during visit 1 (preliminary/baseline), they will receive an intraarticular injection of UC-derived WJ (GeneXSTEM^TM^) by the site PI during Visit 2.1 (procedure).

### Assessment points

Assessments for the study period will start at visit 1 (preliminary/baseline) which includes a thorough review of the patient’s inclusion/exclusion criteria and proper documentation of the informed consent form prior to participation. Once these steps are met, participant’s demographic information, medical history, and baseline case report forms (CRFs) such as NPRS, KOOS, 36-item short-form survey (SF-36), and Single Assessment Numeric Evaluation (SANE) will be collected. Baseline plain radiography (Standing AP, Flexion PA (Rosenberg method), Lateral, and Merchant views) for OA grading using the KL scale will be obtained. Participants will also undergo a T2-weighted MRI and receive a Magnetic Resonance Observation of Cartilage Repair Tissue (MOCART) score. Additionally, a comprehensive metabolic profile, liver function tests, complete blood count, markers of inflammation (C-reactive protein, erythrocyte sedimentation rate), T,B and NK cell lymphocyte subsets, and serum IgG, IgA, IgM, and IgE levels will be collected. At visit 2.2, immediately after the injection procedure, and at visits 3 (24-h follow-up) and 4 (48-h follow-up), NPRS will be collected. During visits 5 (1-week follow-up) and 6 (6-week follow-up), CRFs (NPRS, KOOS, 7-point Likert scale, and SANE) will be collected. Participants will also undergo a PE and have their comprehensive metabolic profile, liver function tests, complete blood count, markers of inflammation (C-reactive protein, erythrocyte sedimentation rate), T,B and NK cell lymphocyte subsets, and serum IgG, IgA, IgM, and IgE levels collected. During visits 7 (3-month follow-up) and 8 (6-month follow-up), participants will undergo the same process as well as have plain radiographs (standing AP, flexion PA (Rosenberg method), lateral and merchant views) taken. During the participants’ final visit, visit 9 (1-year follow-up), the same process as in visits 7 and 8 will be undertaken with an additional T2-weighted MRI for a MOCART score. Participants will have opportunities to report any AEs at each visit or at any time during the study.

### Endpoints

#### Primary endpoint

1. To determine the safety of intraarticular UC-derived WJ formulation (GeneXSTEM^TM^).

#### Secondary endpoints

1. To assess change in patient-reported outcome measures, NPRS and KOOS, from baseline to various follow-up time points.
2. To assess cartilage formation via MOCART at the 1-year time point and compare if from baseline.
3. To assess patient satisfaction using SF-36, 7-point Likert scale and Single Assessment Numeric Evaluation (SANE).

### Sample size and statistical analysis

Descriptive statistics will be computed for all study variables. Continuous variables will be described with measures of central tendency (mean, median) and dispersion (range, standard deviation). Categorical variables will be summarized as frequencies and percentages. Comparisons between categorical variables will be compared with the chi-square test; continuous variables will be compared with Student’s *t* test or non-parametric equivalents. Paired continuous data will be assessed with a paired *t* test or Wilcoxon signed rank test, depending on distribution. Paired categorical data will be assessed with McNemar’s test. For the longitudinal data, a mixed-model repeated measures analysis will be used to examine the between subject factors and the within subject factor of time (baseline, visit 1, visit 2, etc.), as well as their interaction, on the outcome variables of interest. Post hoc tests with corrections for multiple comparisons will be run to determine where significance lies. *P* values < 0.05 will be considered statically significant.

### Data collection and handling

The PI will maintain all source documents. The data will be duplicated on paper study CRFs, and the PI will secure original data in order to be made available to the sponsor and study monitors. Hard copies of CRFs and media will be stored in a secure location and maintained by the PI for a period of 7 years. CRFs will be available for initial inspection for omitted data, data inconsistencies, illegible data, and deviations by the study monitors.

The PI will be responsible for submitting data and reports as follows:

- AEs: in an ongoing basis. This will be reported in the proper section of the CRF.
- Severe AEs: report within 24 h of knowledge of event to sponsor and report to IRB within 5 days as per their regulations.
- Deviations, exceptions, violations of protocol: report to sponsor within 5 days and report to IRB per their regulations.
- Protocol progress report: provide a copy to sponsor and IRB as per regulations.
- Study closure report: provide a copy to sponsor and IRB as per regulations.

### Quality control and assurance

Documents and data will be produced and maintained to ensure control and protection of the patient’s privacy. The protocol, CRFs, and medical records will be available for access by the Sponsor, study monitors, and representatives of regulatory authorities. All attempts will be made to preserve the patient’s privacy and confidentiality.

## Discussion

OA is the most common joint disorder in the USA. It causes significant pain and loss of function for patients and leads to significant strain on the healthcare system [1]. The knee is the most commonly affected joint, and current treatments of OA focus on decreasing pain, increasing function, and improving quality of life. These treatments, however, fail to effectively resolve the underlying pathophysiological processes involved in OA or regenerate diseased cartilage. This is one of the many reasons why the field of regenerative medicine and the use of biologics including UC-derived WJ have grown so rapidly.

This trial will be one the first to evaluate the safety and efficacy of intraarticular UC-derived WJ with patients with grade II or III knee OA. We anticipate that the intraarticular injection of UC-derived WJ is safe, and participants will show an improvement in their overall satisfaction, pain, function, and quality of life. We also hypothesize that cartilage formation over a period of 1 year compared to the baseline visit will improve. Positive outcomes from this study will also lay the foundation for a large placebo-controlled trial of intraarticular UC-derived WJ for symptomatic knee OA.

## Data Availability

The datasets used and/or analyzed during the future study will be available from the corresponding author on reasonable request.

## Abbreviations

AEs: Adverse events
ANOVA: Analysis of variance
CKs: Cytokines
CRFs: Case report forms
EVs: Extracellular vesicles
GFs: Growth factors
HA: Hyaluronic acid
KL: Kellgren-Lawrence scale
KOOS: Knee Injury and Osteoarthritis Outcome Score
MOCART: Magnetic Resonance Observation of Cartilage Repair Tissue
NPRS: Numeric pain rating scale
OA: Osteoarthritis
PI: Principal investigator
SANE: Single Assessment Numeric Evaluation
TKR: Total knee replacement
UC-derived WJ: Umbilical cord-derived Wharton’s Jelly
